# Ameliorative Effect of Allopurinol on Vascular Complications of Insulin Resistance

**DOI:** 10.1155/2015/178540

**Published:** 2015-02-15

**Authors:** Hany M. El-Bassossy, Ahmed A. Elberry, Ahmad Azhar, Salah A. Ghareib, Abdulrahman M. Alahdal

**Affiliations:** ^1^Department of Pharmacology and Toxicology, Faculty of Pharmacy, King Abdulaziz University, Jeddah 21589, Saudi Arabia; ^2^Department of Pharmacology, Faculty of Pharmacy, Zagazig University, Zagazig 44519, Egypt; ^3^Department of Clinical Pharmacy, Faculty of Pharmacy, King Abdulaziz University, Jeddah 21589, Saudi Arabia; ^4^Department of Pharmacology, Faculty of Medicine, Beni Suef University, Beni Suef 62511, Egypt; ^5^Department of Pediatrics, Faculty of Medicine, King Abdulaziz University, Jeddah 21589, Saudi Arabia

## Abstract

The aim of the current study was to evaluate the possible protective effect of allopurinol (Allo) on experimentally induced insulin resistance (IR) and vascular complications. Rats were divided into four groups: control, IR, allopurinol-treated IR (IR-Allo), and allopurinol-treated control (Allo). IR was induced by adding fructose and high fat, high salt diet for 12 weeks. The results showed that Allo has alleviated the increased level of TNF-*α* and the systolic, diastolic, mean, and notch pressure observed in IR with no change in pulse pressure. In addition, Allo decreased the heart rate in the treated group compared to IR rats. On the other hand, it has no effect on increased levels of insulin, glucose, fructosamine, or body weight gain compared to IR group, while it increased significantly the insulin level and body weight without hyperglycemia in the control group. Moreover, Allo treatment ameliorated increased level of 4HNE, Ang II, and Ang R1. In conclusion, the results of the current study show that Allo has a protective effect on vascular complications of IR which may be attributed to the effect of Allo on decreasing the TNF-*α*, 4HNE, Ang II, and Ang R1 as well as increasing the level of insulin secretion.

## 1. Introduction

Insulin resistance (IR) is associated with obesity and characterized by its metabolic consequences, including hyperglycemia, dyslipidemia, and hypertension [1]. These metabolic consequences are known collectively as “metabolic syndrome” (MetS) and are potent risk factors for adverse clinical outcomes [2]. IR has been found to be associated, directly and indirectly, with cardiovascular complications, including atherosclerosis that may lead to myocardial infarction and stroke [3].

Moreover, obesity is associated with increased production of proinflammatory adipokines, including monocyte chemoattractant protein-1, interleukin-6 (IL-6), and tumor necrosis factor-*α* (TNF-*α*). They contribute to chronic, low-grade inflammation and play a pivotal role in the development of insulin resistance [4, 5].

Fructose intake has been directly linked to hyperuricemia [6], which may result in obesity and MetS [7, 8]. The mechanism by which uric acid (UA) induces the development of MetS may be due to UA inhibition of nitric oxide synthase and induction of endothelial dysfunction [9, 10]. Recent evidence supports the concept that hyperuricemia itself can be a significant and independent cardiovascular risk factor [11]. Lowering UA in fructose-induced MetS has been found to be associated with lowering BP, improving insulin sensitivity, and reducing hypertriglyceridemia, through improving endothelial and adipocyte dysfunction [12]. Therefore, the aim of the current study was to evaluate the effect of allopurinol, as a xanthine oxidase enzyme inhibitor reducing the level of UA, on insulin resistance induced experimentally in rats and its possible protective effect on the cardiovascular complications.

## 2. Materials and Methods

### 2.1. Drugs and Chemicals

The following drugs and chemicals were used: allopurinol and urethane (Sigma-Aldrich, St. Louis, MO, USA). Allopurinol and urethane were dissolved in distilled water.

### 2.2. Animals

Male Wistar rats weighing 120–140 g (King Fahd Center for Medical Research, King Abdulaziz University, Saudi Arabia) were housed in clear polypropylene cages (four rats per cage) and kept on equal durations of the dark-light cycle, under constant environmental conditions. Rats received normal rodent pellet diet and water* ad libitum*. Experimental protocol was approved by the Research Ethics Committee of the Faculty of Medicine, King Abdulaziz University.

### 2.3. Study Protocol

Rats were randomly divided into four experimental groups (eight animals each) as follows: control, insulin resistant (IR), allopurinol-treated insulin resistance (IR-Allo), and allopurinol-treated control (Allo). IR was induced by adding fructose (10%) to everyday drinking water and feeding rats on high fat, high salt diet (16% crude protein, 28.2% crude fat, 2.8% crude fiber, 4.8% Ash, and 3.4% salt) for 12 weeks, while control animals receive tap water and standard diet (20% crude protein, 4% crude fat, 3.5% crude fiber, 6% Ash, and 0.5% salt). Allopurinol (20 mg·kg^−1^·day^−1^) was daily administered by dissolving in drinking water (90–110 mg/L) depending upon water consumption [13]. Drinking water was measured every week and allopurinol concentration in drinking water was readjusted.

At the end of the study, animals were fasted 8 hours. The blood glucose level was determined. Then the rats were anesthetized by intraperitoneal injection of urethane (1.5 g·kg^−1^) and the invasive BP was recorded. Finally, venous blood was withdrawn and allowed to coagulate for 30 min at 4°C and then was centrifuged (3000 ×g, 4°C, 20 min) to separate serum. Serum was divided into aliquots and stored at −20°C till being analyzed for insulin, TNF-*α*, adiponectin, and lipid profile.

### 2.4. Serum Analysis

Glucose was determined in tail blood by a glucose meter (Bionime GmBH) using noble metal electrode strips. Serum insulin level as well as fructosamine was measured by enzyme-linked immunosorbent assay (ELISA, Millipore, Billerica, MA, USA) that uses a plate coated with monoclonal anti-rat insulin antibodies.

The homeostasis model assessment of insulin resistance (HOMA-IR) (IR index) was calculated using the serum nonfasting glucose and insulin levels according to the equation of Matthews et al. [14]: HOMA-IR = glucose concentration (mmol/L) × insulin (*μ*U/L)/22.5.

Serum levels of UA, triglycerides (TG), total cholesterol (TC), and high density lipoprotein cholesterol (HDL-C) were determined using the ELITech assay kit (ELITech, Laindon, Essex, France). The low density lipoprotein cholesterol (LDL-cholesterol) was computed using the Friedewald [15] equation: total cholesterol − (HDL-C + 1/5TG).

### 2.5. Blood Pressure (BP) and Heart Rate (HR) Measurement

Invasive BP was continuously recorded for 10 minutes by microtip catheter (Millar, Bella Vista, Australia) inserted in the thoracic aorta through the right carotid artery. The microtip catheter was connected to Power Lab Data Interface Module connected to a PC running LabChart professional software (v7.3, ADI Instruments, Bella Vista, Australia) containing BP module. The BP module detects and calculates different BP parameters like systolic BP, diastolic BP, mean BP, notch pressure, heart rate, cycle duration, ejection duration, and diastolic duration.

### 2.6. Measurement of TNF-*α* and Adiponectin

Serum TNF-*α* and adiponectin levels were determined by ELISA using Quantikine kit (R&D systems, Minneapolis, MN, USA) using rat TNF-*α* or rat adiponectin and antibodies raised against the rat TNF-*α* or rat adiponectin, respectively.

### 2.7. Immunofluorescent Studies

Immunofluorescence staining of 4-hydroxy-2-nonenal (4-HNE), Ang II, Ang R1, and collagen protein expression in rat paraffin embedded aortic sections (5 *μ*m) was carried out according to the method used in our previous works [16–18]. Fixed aortic tissue section slides were deparaffinized in xylene and rehydrated in ethanol and distilled water. Then, perforation is carried out by incubation with methanol at −20°C for 30 min followed by washing with distilled water. Epitopes were retrieved (antigen retrieval) in citrate buffer for 30 min at 95°C followed by washing with PBS. Slides were then immediately transferred into a humidity chamber. Nonspecific binding sites were blocked (PBS containing 5% NGS, 1% BSA, and 0.1% Triton) at room temperature for 1 h. After the blocking, sections were washed (3× 5 min) with PBS. Aortic sections were then incubated with the intended primary antibody diluted in blocking buffer at 4°C overnight. The sections were then washed (3× 5 min) with PBS followed by incubation with a fluorescent conjugated secondary antibody (dilution 1 : 200 in blocking buffer) for 1 h in dark. Then sections were washed (3× 5 min) with PBS and slides were dried and mounted with Prolong lasting mounting media. The slides were stored in dark overnight before examination with Zeiss LSM 780 confocal microscope (Carl Zeiss, Gottingen, Germany) at excitations (488 and 561 nm) and filters (497–542 and 596–655 nm). Images were acquired with identical acquisition parameters, with minimum excitation and gain. Quantitative comparisons of images fluorescence were made with ImageJ software (National Institute of Health, Bethesda, MD, USA). For printing purposes, the level of the confocal images was equally adjusted after the fluorescence quantifications were carried out on unmanipulated images. Sections treated with the secondary antibody alone did not show specific staining while incubating the primary antibody with the blocking peptide significantly reduced the signal. The used primary antibodies were rabbit polyclonal anti-Ang II (1 : 2000, Phoenix Pharmaceuticals Inc., Burlingame, CA, USA), mouse monoclonal anti-angiotensin II type 1 receptor (1 : 133, Abcam, Cambridge, MA, USA), mouse monoclonal anti-collagen type I (1 : 1000, Abcam), and rabbit polyclonal anti-4HNE (1 : 250, Millipore, Billerica, MA, USA). The used secondary antibodies were Alexa Fluor (*λ*_ex_ = 488) conjugated goat anti-mouse and Alexa Fluor (*λ*_ex_ = 594) conjugated goat anti-rabbit (1 : 200, Life Technologies, Grand Island, NY, USA).

### 2.8. Statistical Analysis

Values are expressed as mean ± SEM. Statistical analysis was performed by analysis of variance (ANOVA) followed by Newman-Keuls' post hoc test using a computer based fitness program (Prism 5, GraphPad, CA, USA).

## 3. Results

### 3.1. Effect of Allopurinol on Body Weight Gain

Body weight gain of the IR group was significantly increased (*P* < 0.001) compared to control group. This increase was ameliorated in IR-Allo group but still significantly higher than the control group (*P* < 0.01). Body weight gain was also higher in Allo group (*P* < 0.01) (Table 1).

### 3.2. Effect of Allopurinol on Biochemical Blood Markers and IR Index

**Figure 1:**
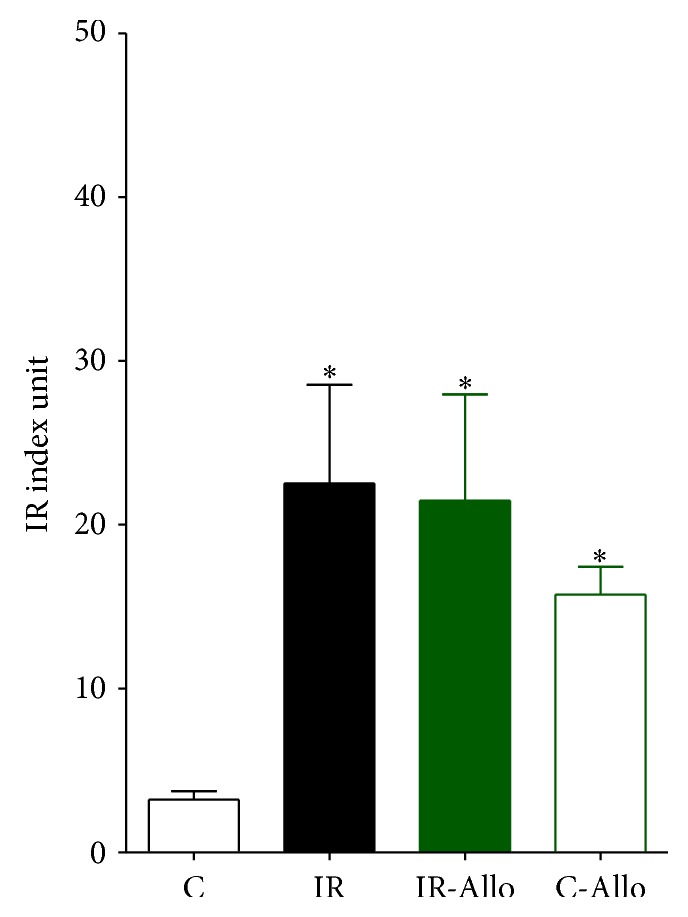
The effect of allopurinol (Allo) treatment on insulin resistance (IR) index.

Regarding UA it was increased in IR group, but this increase was nonsignificant. Treatment with Allo has decreased the level of UA significantly (*P* < 0.01) in IR-Allo group, while there was no effect in Allo group. Fructosamine, fasting blood glucose, and insulin were significantly increased in IR group (*P* < 0.01, *P* < 0.001, and *P* < 0.01, resp.). These failed to be ameliorated by Allo in IR-Allo group. Allo showed no effect on fructosamine or fasting blood glucose in Allo group but showed significant increase (*P* < 0.01) in insulin level compared to control group (Table 1). IR index was increased in all treated groups compared to the control untreated group. This increase was significant in IR and IR-Allo groups (*P* < 0.05) (Figure 1).

### 3.3. Effect of Allopurinol on Lipid Profile

Table 2 revealed that IR model in the current study failed to show any significant changes regarding the fasting lipoprotein profile. However, there was tendency to increase both TG and HDL-C, but this increase was nonsignificant. Allo treatment has decreased nonsignificantly the TG level and increased nonsignificantly the LDL-C level with no effect on TC or HDL-C in either IR-Allo group or Allo group.

### 3.4. Effect of Allopurinol on BP Changes

**Figure 2:**
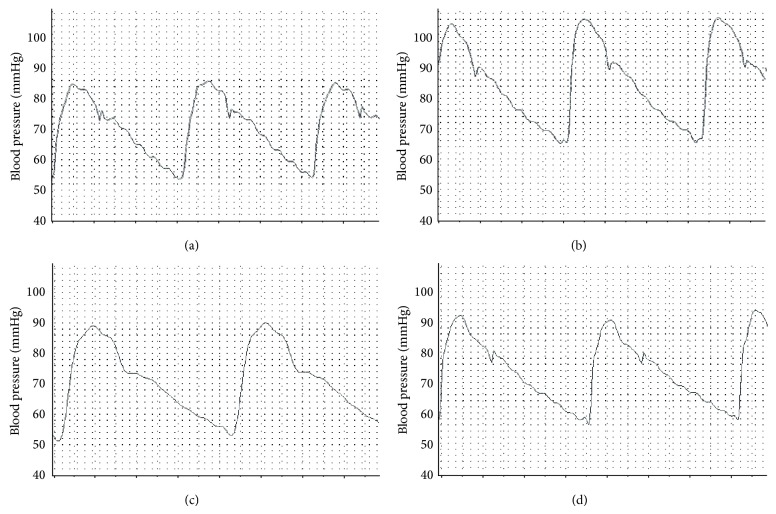
The effect of allopurinol on insulin resistance induced blood pressure changes in BP tracing. Normal control BP (a) shows control systolic and diastolic pressure. IR BP tracing (b) shows increase of both systolic and diastolic BP. IR + allopurinol (c) shows nearly normalized diastolic BP with significant decrease of the elevated systolic BP and increase in the diastolic time. Control + allopurinol (d) shows nearly normal systolic and diastolic BP with minimal increased diastolic time.

**Figure 3:**
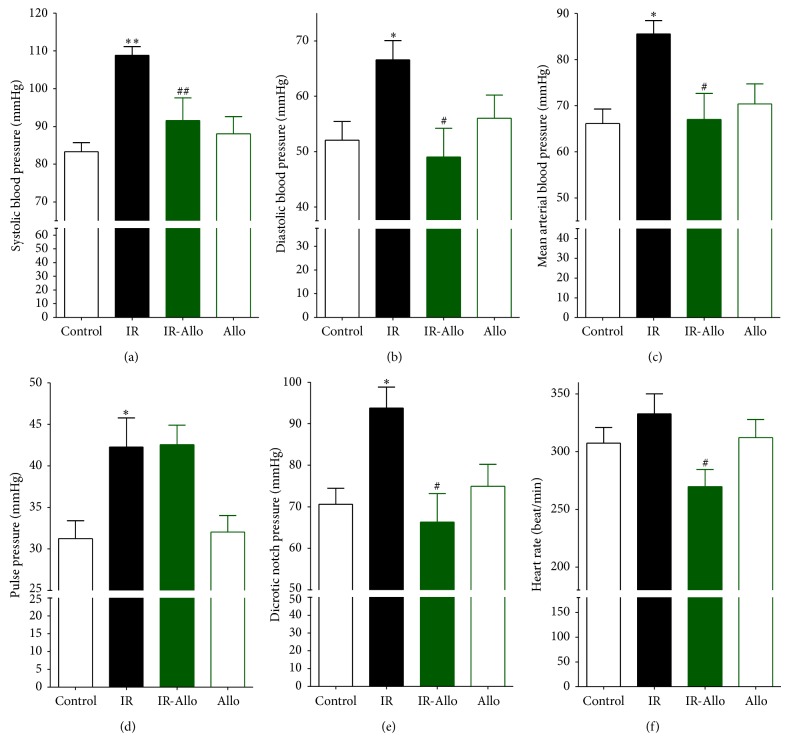
The effect of allopurinol (Allo) treatment on insulin resistance (IR) induced BP changes measured in mmHg and heart rate measured as beats/minute compared to control group (C). (a) Systolic BP, (b) diastolic BP, (c) mean arterial BP, (d) pulse pressure, (e) dicrotic notch pressure, and (f) heart rate. Values are expressed as the mean ± SEM; *N* = 6–8 animals; ^*^ *P* < 0.05 and ^**^ *P* < 0.01 compared with the corresponding control (C) group values; ^#^ *P* < 0.05 and ^##^ *P* < 0.01 compared with the corresponding IR group values, by One-Way ANOVA and Newman-Keuls post hoc test.

BP tracing showed normal BP values including systolic pressure, diastolic pressure, mean arterial pressure, and dicrotic notch pressure as well as normal heart rate in both control group and Allo group (Figures 2 and 3). Moreover, cycle duration in all rats in both control and Allo groups was found to be normal (Table 3). IR has significantly increased systolic (*P* < 0.01), diastolic (*P* < 0.01), mean (*P* < 0.01), pulse (*P* < 0.01), and dicrotic notch (*P* < 0.01) pressures. It also increased the heart rate, but this increase was nonsignificant. Treatment of IR with Allo has significantly decreased the elevated systolic (*P* < 0.05), diastolic (*P* < 0.05), mean (*P* < 0.05), and dicrotic notch (*P* < 0.05) pressures with no effect on pulse pressure. Moreover, Allo has decreased the heart rate compared to control and IR groups (Figures 2 and 3).

Regarding the cycle duration IR has no significant changes, including total cycle duration, ejection duration, diastolic duration, and time to peak. In addition, Allo has no effect in all cycle durations except for the ejection duration where it has significantly (*P* < 0.05) increased compared to the control group.

### 3.5. Effect of Allopurinol on TNF-*α* and Adiponectin

**Figure 4:**
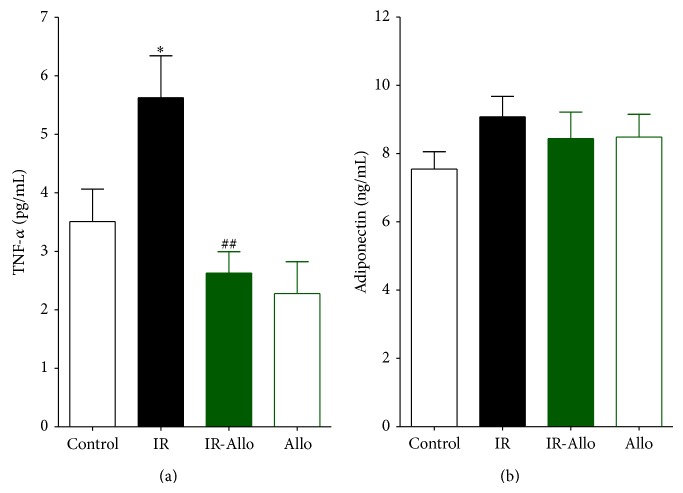
The effect of allopurinol (Allo) treatment on insulin resistance (IR) induced TNF-*α* (a) and adiponectin (b), where they are measured in pg/mL. Values are expressed as the mean ± SEM; *N* = 6–8 animals; ^*^ *P* < 0.05, ^**^ *P* < 0.01, and ^***^ *P* < 0.001, compared with the corresponding control group values; ^#^ *P* < 0.05 and ^##^ *P* < 0.01 compared with the corresponding IR group values, by One-Way ANOVA and Newman-Keuls post hoc test.

TNF-*α* was significantly increased in IR group (*P* < 0.001). Allo treatment was significantly (*P* < 0.01) able to decrease this increased level to the nearly normal control value, while Allo treatment has no effect on Allo group (Figure 4(a)). Regarding adiponectin, IR showed no effect on its level and the treatment with Allo has no effect also in IR-Allo group. Moreover, Allo treatment in Allo group revealed no effect on the level of adiponectin (Figure 4(b)).

### 3.6. Immunofluorescent Results

**Figure 5:**
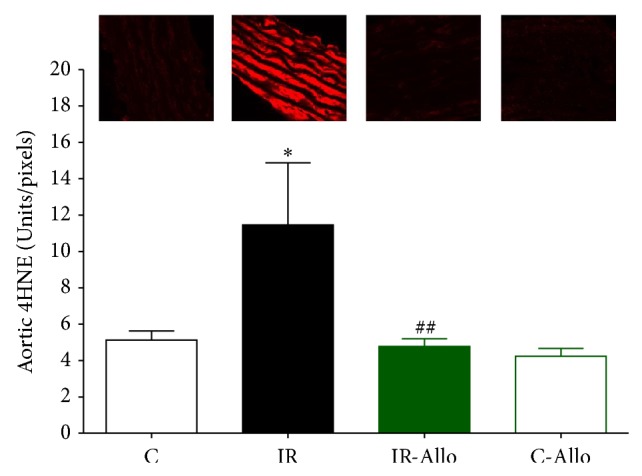
The effect of allopurinol (Allo) treatment on insulin resistance (IR) induced 4HNE changes, measured in Units/pixels. Values are expressed as the mean ± SEM; *N* = 6–8 animals; ^*^ *P* < 0.05, compared with the corresponding control group values; ^##^ *P* < 0.01 compared with the corresponding IR group values, by One-Way ANOVA and Newman-Keuls post hoc test.

**Figure 6:**
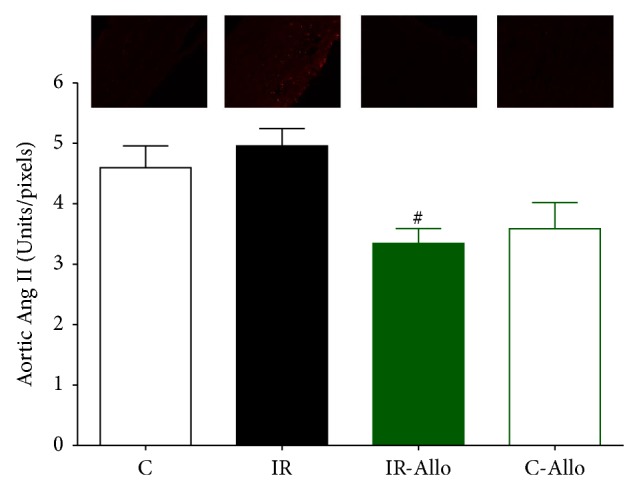
The effect of allopurinol (Allo) treatment on insulin resistance (IR) induced Ang II changes, measured in Units/pixels. Values are expressed as the mean ± SEM; *N* = 6–8 animals; ^*^ *P* < 0.05, compared with the corresponding control group values; ^#^ *P* < 0.05 compared with the corresponding IR group values, by One-Way ANOVA and Newman-Keuls post hoc test.

**Figure 7:**
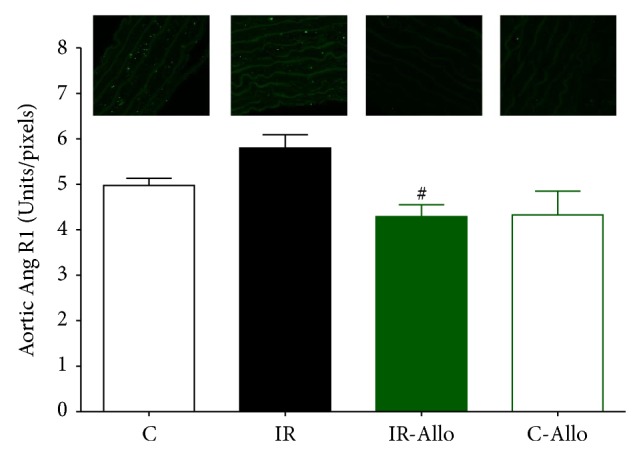
The effect of allopurinol (Allo) treatment on insulin resistance (IR) induced Ang R1 changes, measured in Units/pixels. Values are expressed as the mean ± SEM; *N* = 6–8 animals; ^#^ *P* < 0.05 compared with the corresponding IR group values, by One-Way ANOVA and Newman-Keuls post hoc test.

**Figure 8:**
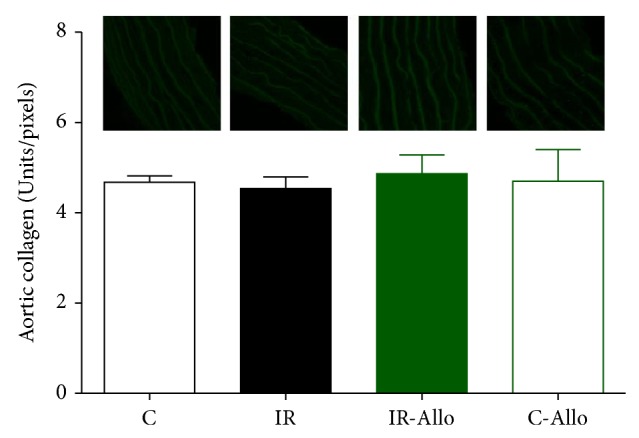
The effect of allopurinol (Allo) treatment on insulin resistance (IR) induced collagen changes, measured in Units/pixels. Values are expressed as the mean ± SEM; *N* = 6–8 animals, with no significant changes by One-Way ANOVA and Newman-Keuls post hoc test.

IR group showed significant increase in 4HNE (*P* < 0.05) compared to control group. Allo treatment significantly (*P* < 0.01) ameliorated this increased level to the normal control values (Figure 5). On the other hand, IR caused no significant changes in Ang II or Ang R1 but Allo treatment caused significant decrease (*P* < 0.05) of both even when compared with normal control values (Figures 6 and 7). Regarding the aortic collagen, no significant changes were observed between all groups (Figure 8).

## 4. Discussion

In the present study, rats fed on fructose and high fat and high salt diet revealed IR evidenced by an increase in both insulin and glucose levels. IR is an important predisposing factor for several clinical disorders, including type 2 diabetes, obesity, dyslipidemia, and hypertension [19]. Although dyslipidemia was not observed in the current study, obesity, increased levels of both glucose and insulin, and hypertension were clearly evident. Moreover, fructosamine, which is an indicator of the average blood glucose concentration over a short-medium period, has increased significantly in the IR model. The prevalence of hyperinsulinemia and IR rise with increasing body mass index and obesity [20], but whether insulin causes these phenomena or is a compensatory response has remained unsettled for decades. The current study showed that Allo has increased the level of insulin without effect on glucose level and associated with increased body weight in control groups. This increase in the body weight may be explained by the allopurinol-induced secretion of insulin observed in the current study with subsequent increase in the glucose uptake in muscles and adipose tissue leading to weight gain with no effect on insulin resistance as the level of glucose was comparable to the normal control group. This finding may need more subsequent studies for accurate and further explanations. Regarding dyslipidemia, a possible explanation for insignificant change in the lipid profile is that fructose-induced hyperuricemia is maximal in the first few hours following ingestion of fructose [21] and is also dose-dependent [22]. Moreover, the small sample size used in the present study may be another explanation for this insignificant change in the lipid profile.

Clinical and experimental studies have assessed the relationship between hyperuricemia and hypertension and found that hyperuricemia might play a double role as a risk factor for hypertension and as a pathological condition enhanced by hypertension itself [11]. Moreover, many studies revealed that IR is a risk factor for cardiovascular disease [3, 23]. Despite the fact that it was nonsignificant, the results of the present study demonstrated that IR caused an increase of the UA level with significant vascular complications including the systolic, diastolic, mean, pulse, and dicrotic notch pressure with no effect on the cycle durations or heart rate. Previous studies found that XO activity is increased by various cytokines, including TNF-*α* [24, 25], which is significantly increased in the current study. Moreover, XO was found to be significantly elevated in a variety of vascular diseases including limb ischemia [26], coronary artery disease [27], and heart failure [28]. Circulating XO binds to glycosaminoglycans on the surface of endothelial cells where it is involved in the pathogenesis of endothelial injury [29, 30]. In addition, our previous study demonstrated that TNF-*α* has an important role in IR and vascular impairment [31]. In contrast, the nonsignificant elevation of UA in the current study may be attributed to the fact that the present model is not the ideal model representing the hyperuricemia using 2% oxanic acid [9]. This may pay attention to the possibility that the effect of Allo is not dependant on decreasing the UA level, but due to effect on other byproducts of purine metabolism by XO as superoxide.

Moreover, hyperglycemia and hyperinsulinemia in IR may reduce arterial wall compliance by promoting plaque growth, vascular smooth muscle cell proliferation, and nonenzymatic glycosylation of vessel wall proteins [32, 33]. It was reported that the vascular endothelium expresses membrane insulin receptors and is a target for the biologic effects of insulin, which stimulate production of nitric oxide (NO) leading to vasodilatation [34]. This effect is blunted in cases of IR. Moreover, hyperglycemia itself increases the release of the vasoconstricting peptide endothelin-1 from cultured endothelial cells, offsetting the vasodilating actions of NO [35].

The results of the current study showed that Allo has alleviated the vascular complications of IR including systolic, diastolic, mean, and dicrotic notch pressures compared to the untreated IR group. The elevations in diastolic and notch BP are largely attributed to the increased peripheral arterial resistance [36], whereas pulse pressure reflects stiffening of large arteries [37]. Pulse pressure was not significantly changed in IR as a result of significant increase in both systolic and diastolic BP in IR group. The present study may provide an explanation for these palliative effects which may be related to the effect of Allo on increasing insulin secretion observed in the Allo control group which in turn have a vasodilator effect on blood vessels [34]. Moreover, previous studies found that Allo improves endothelial dysfunction and prevents the development of arteriosclerosis [38]. Moreover, inhibition of XO by Allo was found to improve endothelium-dependent dilation and reduced superoxide production in isolated coronary arterioles following ischemia reperfusion [39]. Another explanation may be related to the effect of Allo on renal function improvement and increasing glomerular filtration rate as shown by Neal et al. [40].

On the other hand, it was found that Allo treatment decreased the heart rate significantly even when compared to the normal controlled group which is in accordance with the results of Sakabe et al. [41] which revealed that Allo has a protective effect on atrial fibrillation in dogs associated with tachycardia-induced cardiomyopathy. Despite the fact that it was nonsignificant, this bradycardia was associated with increase in the total cycle duration.

Moreover, the current study showed an increased level of TNF-*α* which is an important inflammatory cytokine that is overexpressed in adipose and other tissues in cases of IR and obesity [42]. This is in accordance with many studies which revealed the role of TNF-*α* in the induction of IR and type 2 diabetes [43, 44]. Although there was no significant change in the lipid profile as mentioned before, this elevated TNF-*α* may be responsible for the mild increase in the TG and HDL-C and decreased level of LDL. Previous studies showed that hepatic TG and VLDL production are increased in both human and murine studies after TNF-*α* administration [45, 46]. This may be due to increased hepatic levels of citrate, the rate-limiting enzyme in free fatty acid synthesis [47], or through inhibition of lipoprotein lipase activity [48]. Whereas TNF-*α*-induced changes in TG metabolism are similar in all species, its effect on cholesterol metabolism differs between rodents and primates. Whereas the administration of TNF-*α* in rodents is followed by increase in hepatic cholesterol synthesis and LDL [45, 49], nonhuman primates and humans show either no change or a decrease in serum cholesterol and LDL levels [50]. The mechanisms underlying this species difference are not known. Regarding the elevated HDL-C, it may be related to the inhibitory effect of TNF-*α* on cholesteryl ester transfer protein (CETP) which transfers cholesteryl esters from HDL to TG-rich lipoproteins leading to high levels of HDL-C [51].

Allo treatment significantly alleviated this increased TNF-*α* level nearly to the normal control levels which is in accordance with previous studies that showed the reducing effect of Allo on TNF-*α* [52, 53]. A novel explanation of this decrease in the TNF-*α* evidenced by the results of the current study is that it may not be due to direct effect, but may be due to secondary to the ability of the Allo to increase the level of insulin. Beyond its classic metabolic actions, insulin has also anti-inflammatory effect, decreasing the activity of proinflammatory cytokines including TNF-*α* [54]. Unlike many studies which revealed that, in IR, the levels of leptin and resistin increase while adiponectin, which has insulin-sensitizing and anti-inflammatory effect, decreases [55–57], the current study revealed no significant change in adiponectin level. This may be explained by the finding of Shapiro et al. [58] who reported that rats fed fructose* ad libitum* chronically become leptin resistant with a compensatory increase in adiponectin.

In addition, the results of the present study showed that treatment with Allo has ameliorated and decreased the increased level of 4HNE that was observed in IR group. It was found that 4HNE is a biomarker aldehyde of oxidative stress which readily binds covalently to nucleophilic residues of proteins, peptides, phospholipids, and nucleic acids and thereby exhibits cytotoxic effects. 4HNE can also modulate signaling pathways involved in cell proliferation, fibrosis, apoptosis, and inflammation, which are all hallmarks of cardiovascular diseases [59–61]. Moreover, the current study revealed that Allo treatment decreased significantly Ang II and Ang R1. Angiotensin II directly causes vasoconstriction, increases the release of antidiuretic hormone via release of vasopressin, thus causing the reabsorption of water at the level of the kidneys, and triggers release of aldosterone from the adrenal cortex, causing the kidney to reabsorb sodium [62]. It is assumed that the current study reveals that the ameliorative effect of Allo treatment is not related to its effect on UA and may be related to other mechanisms as the UA in the present model of IR was not significantly increased compared to the normal control group.

In conclusion, the results of the current study show that Allo has a protective effect on vascular complications of IR which may be attributed to the effect of Allo on decreasing the level of TNF-*α*, 4HNE, Ang II, and Ang R1 as well as increasing the level of insulin secretion regardless of the effect of associated dyslipidemia.

## Figures and Tables

**Table 1:** The effect of allopurinol (Allo) treatment on insulin resistance (IR) induced body weight changes and blood biochemical markers including uric acid, fructosamine, fasting blood glucose, and insulin compared to control group.

	Body weight gain % from baseline	Uric acid (pg/mL)	Fructosamine (*µ*mol/L)	Fasting blood glucose (mg/dL)	Insulin (ng/mL)
Control group	39.81 ± 4.913	0.95 ± 0.15	46.63 ± 3.75	73.29 ± 2.86	0.782 ± 0.13
IR group	157.5 ± 29.35^***^	1.18 ± 0.08	71.47 ± 6.05^**^	118.30 ± 9.70^***^	3.149 ± 0.31^**^
IR-Allo Group	115.5 ± 16.60^**^	0.55 ± 0.10^##^	61.33 ± 1.92^**^	112.20 ± 0.86^***^	3.322 ± 0.49^**^
Allo group	143.6 ± 8.392^**^	0.97 ± 0.03	52.00 ± 3.29	85.00 ± 8.77	2.822 ± 0.32^**^

Values are expressed as the mean ± SEM; *N* = 6–8 animals.

^*^ *P* < 0.05, ^**^ *P* < 0.01, and ^***^ *P* < 0.001, compared with the corresponding control group values; ^#^ *P* < 0.05 and ^##^ *P* < 0.01 compared with the corresponding IR group values, by One-Way ANOVA and Newman-Keuls post hoc test.

**Table 2:** The effect of allopurinol (Allo) treatment on insulin resistance (IR) lipoprotein profile including total cholesterol (TC), low density lipoprotein (LDL), triglycerides (TG), and high density lipoprotein (HDL-C) compared to control group.

	TG (mg/dL)	TC (mg/dL)	LDLC (mg/dL)	HDL (mg/dL)
Control group	45.85 ± 2.55	50.15 ± 2.42	29.08 ± 6.41	13.75 ± 2.85
IR group	55.17 ± 6.89	48.50 ± 4.29	16.94 ± 4.01	21.19 ± 2.58
IR-Allo group	45.36 ± 3.23	53.72 ± 4.56	23.04 ± 3.67	21.07 ± 2.50
Allo group	47.04 ± 2.94	51.49 ± 2.13	23.10 ± 2.10	18.99 ± 1.27

Values are expressed as the mean ± SEM; *N* = 6–8 animals, without any significant differences by One-Way ANOVA and Newman-Keuls post hoc test.

**Table 3:** The effect of allopurinol (Allo) treatment on insulin resistance (IR) induced BP cycle duration changes measured in seconds compared to control group.

	Total cycle duration	Ejection duration	Diastolic duration	Time to peak
Control group	0.196 ± 0.007	0.069 ± 0.002	0.127 ± 0.007	0.040 ± 0.003
IR group	0.190 ± 0.011	0.062 ± 0.004	0.123 ± 0.01	0.028 ± 0.002
IR-Allo group	0.220 ± 0.012	0.104 ± 0.013^#^	0.118 ± 0.010	0.038 ± 0.002
Allo group	0.194 ± 0.009	0.066 ± 0.005	0.129 ± 0.007	0.036 ± 0.004

Values are expressed as the mean ± SEM; *N* = 6–8 animals.

^*^ *P* < 0.05, ^**^ *P* < 0.01, and ^***^ *P* < 0.001, compared with the corresponding control (C) group values; ^#^ *P* < 0.05, ^##^ *P* < 0.01, and ^###^ *P* < 0.001 compared with the corresponding IR group values, by One-Way ANOVA and Newman-Keuls post hoc test.
